# Conservative Treatment of Dentigerous Cyst by Marsupialization in a Young Female Patient: A Case Report and Review of the Literature

**DOI:** 10.1155/2018/7621363

**Published:** 2018-06-28

**Authors:** Layal Ghandour, Hisham F. Bahmad, Samar Bou-Assi

**Affiliations:** ^1^Department of Orthodontics and Dentofacial Orthopedics, Faculty of Dentistry, Lebanese University, Hadath, Lebanon; ^2^Department of Anatomy, Cell Biology, and Physiological Sciences, Faculty of Medicine, American University of Beirut, Beirut, Lebanon; ^3^Division of Orthodontics and Dentofacial Orthopedics, Department of Otolaryngology-Head and Neck Surgery, Faculty of Medicine, American University of Beirut Medical Center, Beirut, Lebanon

## Abstract

Dentigerous cysts (DCs) are the most prevalent developmental odontogenic cysts that occur in middle-aged individuals. They frequently originate from the epithelial remnants of tooth-forming organs. Hereby, we present a case of a 13-year-old young female patient presenting with DC that was treated successfully by marsupialization. The patient's chief complaint was the crowding of the anterior teeth. Clinical examination showed that the patient had all her permanent teeth present with a retained mandibular left second primary molar that was previously treated by pulpectomy. The radiographic examination revealed a unilocular radiolucent lesion with well-defined margins associated with a mesially-tipped unerupted mandibular left second premolar. The differential diagnosis confirmed that the lesion was a DC. The treatment consisted of surgical removal of the DC to allow proper eruption of the permanent tooth and to prevent the lesion from becoming an aggressive one causing gross expansion of bone with subsequent facial asymmetry, pain, displacement of teeth, and root resorption. A removable acrylic obturator was delivered to the patient keeping the path clear and guiding the eruption of the premolar until fully erupted.

## 1. Introduction

Odontogenic cysts are a group of common lesions occurring in the maxilla and mandible and are of the main causes of the destruction of these bones [1]. A dentigerous cyst (DC) is a cyst that is characterized by the attachment to the crown of an unerupted tooth [2]. The exact pathogenesis of DC is obscure; however, most authors believe that this cyst is of a developmental origin from tooth follicles.

The DCs are seldom discovered in young individuals since they frequently occur in individuals between 20 and 40 years of age. These cysts are discovered unexpectedly on routine radiographic examination since DCs are asymptomatic unless after an infection [3, 4].

The aim of the present study is to present a clinical case of a DC in a young female patient that was successfully treated by marsupialization.

## 2. Case Presentation

A 13-year-old female patient was presented for consultation to the Department of Orthodontics and Dentofacial Orthopedics, School of Dentistry, Lebanese University. Her chief complaint was the crowding of her anterior teeth. Her medical and dental histories were noncontributory, and the patient did not mention any previous or recent habit. On physical examination, no swelling or tenderness was documented.

**Figure 1:**
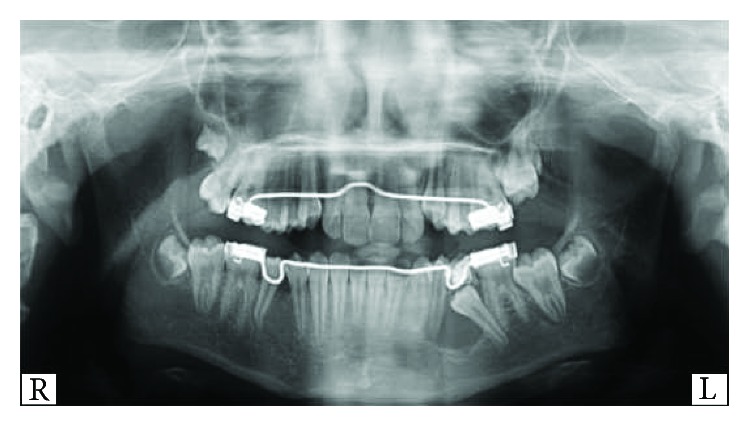
Orthopantomogram (OPG) showing a radiolucent lesion at the mandibular left side (second premolar region).

**Figure 2:**
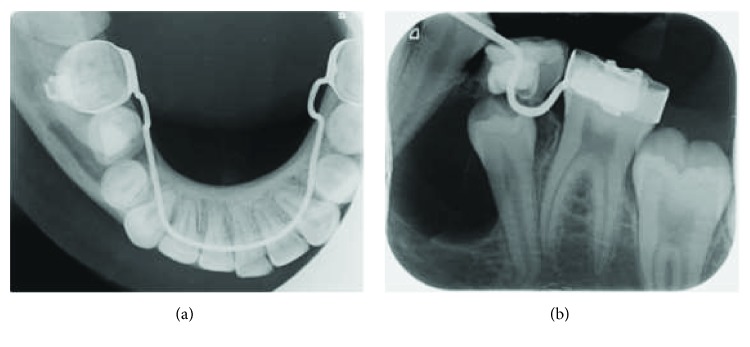
(a) Occlusal radiograph to further investigate the well-defined radiolucent lesion. (b) Periapical radiograph to further investigate the well-defined radiolucent lesion.

Upon clinical examination, the patient had all her permanent teeth and a retained mandibular left second primary molar. Radiographic records consisted of an orthopantomogram, a lateral cephalogram, a posteroanterior cephalogram, and a hand wrist radiograph. The orthopantomogram revealed a well-defined radiolucent lesion on the mandibular left side surrounding the unerupted mandibular left second premolar which appeared to be mesially tipped below the retained primary second molar. The root of the adjacent premolar was included in the lesion but did not reveal any root resorption (Figures 1 and 2).

**Figure 3:**
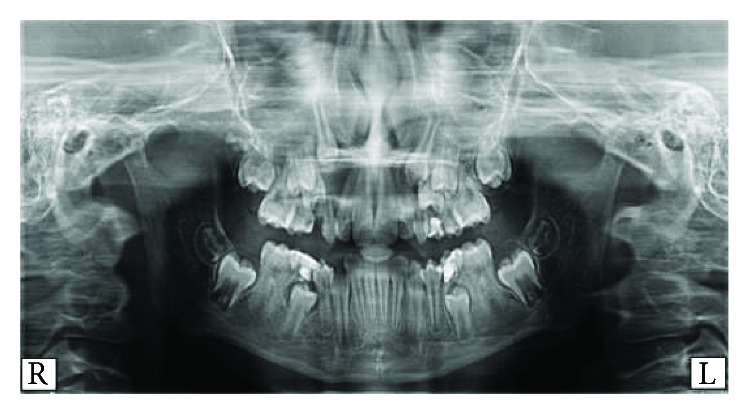
Older orthopantomogram (OPG) showing the absence of any radiolucent lesion at the mandibular left side (second premolar region).

Going back to the old orthopantomogram (OPG) of the patient collected by the Pediatrics Department, it was noted that no lesion was visible at that time (Figure 3). The patient was referred to the Oral Pathology Department in order to obtain a meticulous diagnosis concerning the radiolucent lesion that was detected on the orthopantomogram during the initial diagnosis. The differential diagnosis for the lesion included a DC, an odontogenic keratocyst, and an ameloblastoma. Histologically, a thick epithelial lining with rete ridges was present. Moreover, chronic inflammatory cellular infiltration appeared in the capsule of the cyst. All these findings confirmed that the diagnosed cyst is a DC. The major objective of initiating the treatment as early as possible in this patient was to hinder the progression of the DC prohibiting its destructive consequences. Moreover, the aim of initiating a nonaggressive (marsupialization) treatment was to save the involved tooth, allowing its healthy eruption.

Several treatment modalities of a DC have been reported in the literature ranging from marsupialization to enucleation. The common treatment for DC is enucleation followed by extraction of the involved tooth. When the cyst is large, the first approach is marsupialization to decrease the size of the osseous defect and then enucleation and tooth extraction are performed afterwards [5–8].

Enucleation is the chosen treatment plan whenever the cyst is small and saving the involved tooth is impossible. This treatment modality should be avoided in large cysts since it is usually accompanied by facial, esthetic, and functional defects [9].

Other approaches are considered when the cyst is small and when the patient is young. In cases of enlarged follicles of impacted canines, exposing the affected tooth surgically and the traction of the tooth by orthodontic means leads to the cessation of the cystic lesion and the preservation of the affected tooth [10].

**Figure 4:**
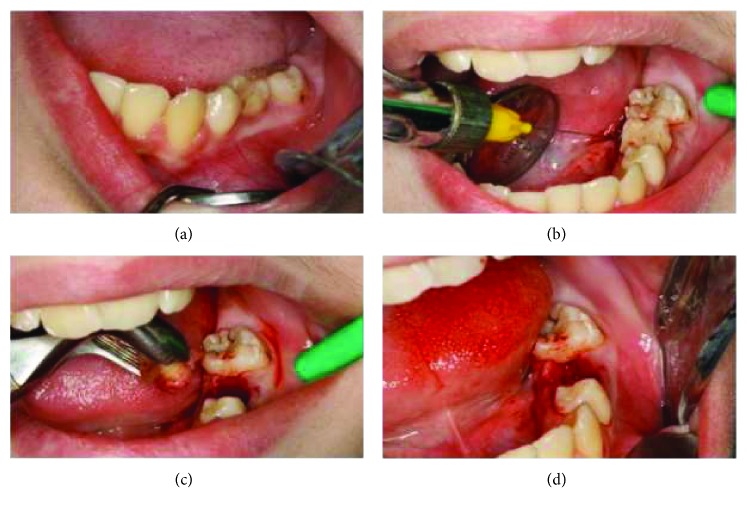
Marsupialization of the cystic lesion after extraction of the deciduous second molar, revealing retraction to visualize the surgical site at first (a), then anesthetizing the site (b), followed by extraction of the primary second molar and drainage (c), and finally opening of the surgical site after marsupialization (d).

Taking into consideration that the diagnosed cyst can potentially become an aggressive lesion with a bone destructive ability that might lead to loss of the involved teeth, root resorption, pain, and facial asymmetry, and considering the age of the patient, a primary approach by marsupialization was initiated (Figure 4).

**Figure 5:**
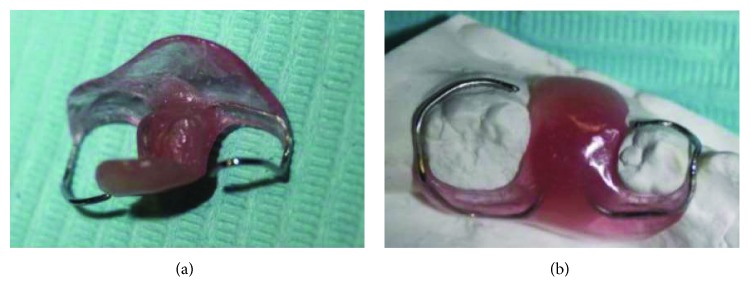
The acrylic resin obturator to maintain the surgical opening during healing.

After the surgery, the patient was instructed to wear the acrylic resin obturator that was previously fabricated to maintain the surgical opening during healing and assure success of the surgery (Figure 5). The patient was asked to eat with the obturator in place and remove it only for cleaning. Follow-up appointments were scheduled every three months postsurgery to assure the eruption of the second premolar and the absence of any recurrence.

**Figure 6:**
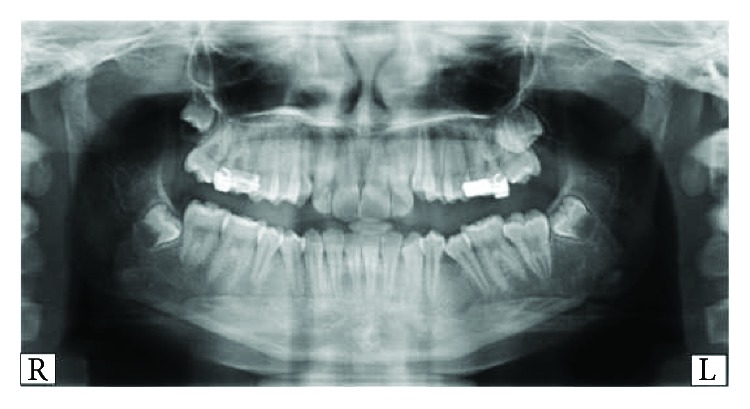
Orthopantomogram (OPG) showing the absence of any radiolucent lesion and the successful eruption of the mandibular left second premolar.

**Figure 7:**
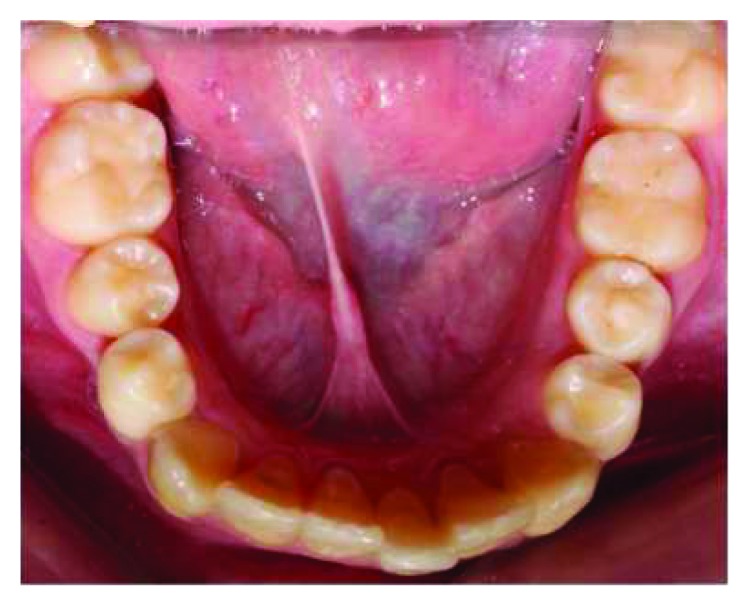
Intraoral photo postmarsupialization with the mandibular left second premolar completely erupted on the arch.

The postsurgical orthopantomogram (OPG) that was taken 6 months after the surgery revealed the absence of any radiolucent lesion and the successful eruption of the mandibular left second premolar (Figures 6 and 7).

## 3. Discussion

Dentigerous cysts (DCs) have been reported extensively in the literature. The exact cause of this cyst is still unknown, but many theories are proposed. The “intrafollicular theory” suggests that a DC is a consequence of fluid accumulation between the outer and inner surfaces of the epithelium. This accumulation occurs during the formation of the crown. The second theory is the “enamel hypoplasia theory”. It suggests the development of the cyst after stellate reticulum degeneration. “Main's theory” suggests that the cyst is a result of the hydrostatic pressure exerted by an impacted tooth on the follicle which results in the separation of the impacted crown from the surrounding follicle.

Some authors believe that a DC is more likely to occur as a result of an inflamed nonvital deciduous tooth during the maturation of the permanent successor. In 1928, Bloch-Jorgensen reported 22 cases of DCs with the cystic wall being in a direct contact with an affected deciduous tooth. Azaz and Shteyer [11], Shaw et al. [12], and many others also pointed out the same inflammatory cause of the cyst.

In the case described in this report, the most probable cause of the DC could be the nonvital deciduous second molar. After a thorough inspection of the radiographic file of the patient, one can notice the absence of radiolucency in the initial orthopantomogram that was taken when the patient was 9 years and 6 months old (Figure 3). Later, after root resorption of the treated root canal of the deciduous molar, the cyst started developing (Figure 2(b)). One explanation is that the nonresorbable material that was used in the pulpectomy of the previously infected deciduous molar acted as a stimulus for the development of the cyst. Pulpectomy, by definition, is the elimination of infected pulp tissues by chemical and mechanical means. After removing the pulp, the canals are filled with a material that supposedly should resorb along with the resorption of the associated root [13].

The incidence of DCs is highest in the second and third decades of life [1, 14]. This cyst is usually rare in the first decade. For this reason, when it comes to diagnosis in young patients, it is usually difficult to state a definitive diagnosis without a pathological and radiographic diagnosis. In the reported case, the definitive diagnosis was confirmed by the Pathology Department at the Lebanese University. The report we received from the Pathology Department included not only the definitive diagnosis of the cyst, but also the differential diagnosis. The differential diagnosis of DCs included odontogenic keratocyst, primordial cyst, ameloblastoma, and ameloblastic fibroma. A comprehensive and meticulous report interpreted the reason for the final diagnosis.

Dentigerous cysts are detected by chance after the patient/dentist realizes that some of his/her primary teeth are still retained. This cyst, because it is usually asymptomatic, is sometimes recognized after it has expanded into the alveolar bone and has led to destruction. Berden et al. found that it is important to choose a safe treatment in young individuals and avoid surgical approaches that lead to esthetic, functional, and psychological problems if facial defects occur. The decompression of large cysts was outweighed when the cyst is large to avoid the previously mentioned drawbacks of enucleation [9]. Whenever these cysts are detected in a late stage, they are usually treated by enucleation followed by the extraction of the involved tooth [15].

Several factors predicting the spontaneous eruption of premolars following marsupialization have been extensively discussed in the literature. For this purpose, many angular and linear values were measured on an orthopantomogram.

Hyomoto et al. measured the cusp depth of the impacted tooth, angle formed between the long axis of the impacted tooth and the bisector of the long axis of adjacent teeth. He also measured the root maturity, the cyst area, and the eruption space available. He found that the smaller the patient, the greater the chance of eruption. A depth of inclusion of 4.4 mm and an angulation of tooth of 20.4° ± 21.8°, 1/2 root formation were associated with spontaneous eruption of impacted teeth. Moreover, he concluded that the space present for eruption did not influence the eruption. In his study, he found that spontaneous eruption of premolars following marsupialization occurred in 72.4% of the cases [16].

Fujii et al. in 2008 analyzed similar factors as Hyomoto et al. [16, 17]. They concluded that spontaneous eruption of impacted teeth associated with DCs after marsupialization is possible in patients less than 10 years old. He added that the depth of inclusion should be smaller than 5.1 mm and that tooth angulation must be smaller than 25°. The space present for eruption should be larger than 1 cm, and the root should be incompletely formed [17].

Yahara et al., in attempt to measure the same factors, found that spontaneous eruption occurred when the mean age was 9.8 years. They also explained that the smaller the depth of inclusion, the better chance of eruption. The angulation that was measured by the abovementioned authors was also measured by Yahara et al. This angle should be close to 60° for the tooth to erupt without any traction [18].

Trying to find the possibility of spontaneous eruption of the affected premolar in this reported case, the same factors were measured. The patient was 13 years old, and depth of inclusion was 3.6 mm. The angle formed between the long axis of the tooth and the bisector of the long axis of both adjacent teeth was 40°. Moreover, three-quarters of the root was formed on the final panoramic radiograph.

Although the age of the patient and the stage of rhizogenesis (root formation) were discouraging, we preferred the conservative approach hoping for the spontaneous eruption of the second premolar.

Conservative treatment of a DC was reported in some cases [19]. The primary objective of such a treatment was to save the involved tooth especially in young patients. This cautious therapy is adopted in children due to the bone reparative capacity in these patients and the potential of teeth with open apices to erupt after the removal of the cyst. When this treatment approach is rendered, the cyst is left opened allowing the cystic drainage and a stent or a removable appliance is placed afterwards. This removable obturator facilitated the drainage of the cyst into the oral cavity, thus relieving the pressure and allowing the tooth to erupt spontaneously. Moreover, being a removable appliance, the oral hygiene was not affected and unwanted inflammation did not occur. In the case reported in the following article, the advocated first line of treatment was a conservative marsupialization that was initiated in attempt to save the premolar. After performing the marsupialization and leaving the surgical site open to the oral cavity, the involved tooth successfully erupted into the dental arch.

## 4. Concluding Remarks

Dentigerous cysts are the most prevalent developmental odontogenic cysts that occur in middle-aged individuals [20]. Although abundant theories had been suggested concerning the cause of this cyst, the precise cause is still unidentified.

Most reported cases of DCs in young individuals were due to infected nonvital deciduous primary molars. The pulpectomy treatment of these nonvital teeth did not cease the enlargement of the cyst. Such a treatment in an attempt to save a primary tooth has led to the extraction of the permanent successor after the cyst has spread and was discovered by chance.

The material used in pulpectomy and the procedure itself should be further studied, and the benefit-to-risk ratio before performing such a treatment should be considered. Moreover, if the practitioner chooses this option, he should insist on the importance of regular checkup appointments. In this way, early detection of abnormal pathological cysts would be possible.

Numerous treatment procedures were discussed in the literature depending on the age of the patient, severity and size of the cyst, and the objectives behind the treatment. A conservative approach should be the first line of treatment whenever possible. The predicting factors for the spontaneous eruption of the permanent successor following marsupialization that were reported in the literature should not be taken as strict guidelines and should not guide the choice of the treatment plan. In the case reported in this paper, marsupialization was performed in order to save the affected tooth. Successful eruption of the mandibular left second premolar was reported.

## Data Availability

A copy of the written consent is available upon request for review of this journal.
